# Synthesis and crystal structures of three Schiff bases derived from 3-formyl­acetyl­acetone and benzyl-, *tert*-butyl- and (*S*)-methyl­benzyl­amine

**DOI:** 10.1107/S205698902300587X

**Published:** 2023-07-11

**Authors:** Jan Henrik Halz, Andreas Hentsch, Christoph Wagner, Kurt Merzweiler

**Affiliations:** a Martin-Luther-Universität Halle-Wittenberg, Naturwissenschaftliche Fakultät II, Institut für Chemie, D-06099 Halle, Germany; Vienna University of Technology, Austria

**Keywords:** crystal structure, enamine, 3-formyl­acetyl­acetone, Schiff base

## Abstract

Treatment of 3-formyl­acetyl­acetone with the amines benzyl­amine, *tert*-butyl­amine and (*S*)-methyl­benzyl­amine led to the formation of the corresponding Schiff bases 3-[(benzyl­amino)­methyl­idene]pentane-2,4-dione, C_13_H_15_NO_2_ (**1**), 3-[(*tert*-butyl­amino)­methyl­idene]pentan-2,4-dione, C_10_H_17_NO_2_ (**2**) and 3-{[(*S*)-benz­yl(meth­yl)amino]­methyl­idene}pentane-2,4-dione, C_14_H_17_NO_2_ (**3**). The mol­ecules of all three compounds exist as enamine tautomers that contain a nearly planar amino-methyl­ene-pentane-2,4-dione core with a strong intra­molecular N—H⋯O hydrogen bridge. The *R* group attached to the enamine N atom has no significant influence on the bond lengths and angles of the amino-methyl­ene-pentane-2,4-dione core. The supra­molecular structures in **1**–**3** are mainly based on weak C—H⋯O hydrogen bonds.

## Abstract 2

The crystal structures of three Schiff bases synthesized from 3-formyl­aceylacetone and different primary amines were determined and compared with simulated gas phase structures based on DFT calculations.

## 1. Chemical context

3-Formyl­acetyl­acetone reacts with primary amines *R*NH_2_ to give enamines with an amino-methyl­ene-pentane-2,4-dione core. The first reference to this type of Schiff base compound dates back to Claisen, who used eth­oxy­lidene­acetyl­acetone as synthetic alternative to 3-formyl­acetyl­acetone. 3-Amino­methyl­ene-pentane-2,4-dione, which may be regarded as the parent compound, was reported as early as 1893 (Claisen, 1893 ▸), and its crystal structure was reported in 2006 (Gróf *et al.*, 2006 ▸ *a*), almost simultaneously with that of the methyl­amino derivative (Gróf *et al.*, 2006 ▸ *b*). In 1966, Wolf & Jäger. successfully used the deprotonation of 3-amino­methyl­ene-pentane-2,4-dione type Schiff bases to generate *β*-imino­enolate chelate ligands, with special focus on tetra­dentate salen-type ligands (Wolf & Jäger, 1966 ▸). In particular, these salen-type ligands have found broad application in the synthesis of Fe^II^ complexes exhibiting spin-crossover effects (Dürrmann *et al.*, 2021 ▸). Moreover, the coordination properties of the imino­enolate ligands are conveniently modified by the introduction of additional donor groups. This is easily done by the reaction of 3-formyl­acetyl­acetone with a suitably functionalized amine, *e.g.* in form of α-amino acids (Hentsch *et al.*, 2014 ▸) or *o*-di­phenyl­phosphinoaniline (Halz *et al.*, 2021 ▸).

In the current communication, we focus on some structural aspects of three derivatives, namely 3-[(benzyl­amino)­methyl­ene]pentane-2,4-dione (**1**), 3-[(*tert*-butyl­amino)­methylen]pent­an-2,4-dione (**2**) and 3-{[(*S*)-methyl­benzyl­amino]­methyl­en}pentane-2,4-dione (**3**). Formally, all three compounds can be derived from 3-[(methyl­amino)­methyl­ene]pentane-2,4-dione by partial or complete replacement of the methyl H atoms with other residues (Me, Ph). It can thus be expected that the main structural differences between compounds **1**–**3** will arise from conformational aspects regarding the orientation of the CH_2_Ph, CH(CH_3_)Ph and C(CH_3_)_3_ moieties with respect to the 3-amino­methyl­ene-pentane-2,4-dione core. In order to get some insight into the differences between solid-state and (theoretical) gas phase structures, compounds **1**–**3** were also characterized by DFT calculations. 
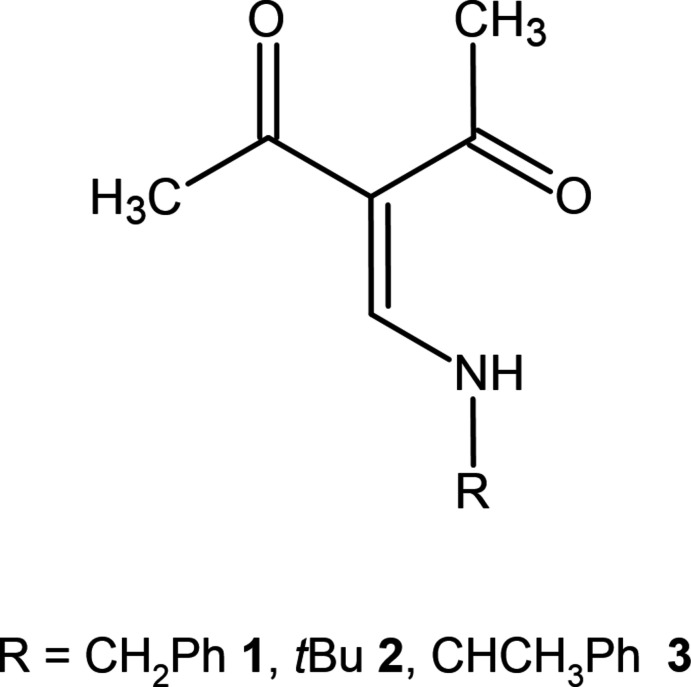


From the synthetic point of view it is worth mentioning that compounds **1** and **2** are also accessible by the eth­oxy­lidene­acetyl­acetone route (Zhou, 1997 ▸). Originally, compound **2** was obtained from a formimidoyl­ation of acetyl­acetone with a substituted imidazole (Ito *et al.*, 1974 ▸).

## 2. Structural commentary

Compounds **1** and **2** crystallize in the monoclinic system, space group *P*2_1_/*c* with *Z* = 4. Compound **3** forms monoclinic crystals in space group *P*2_1_ with *Z* = 4. Both independent mol­ecules in the asymmetric unit of **3** exhibit nearly identical bond lengths and angles.

**Figure 1:**
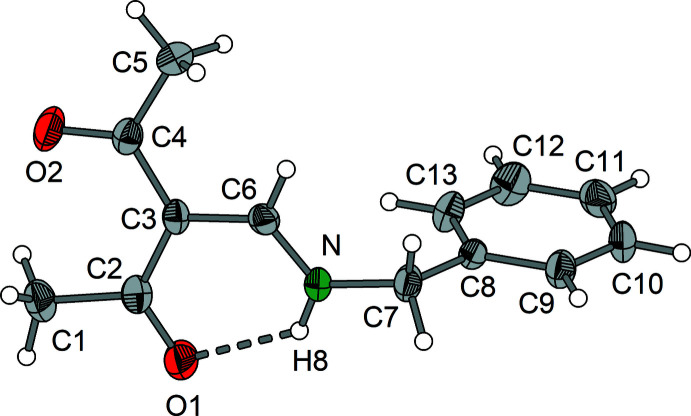
Mol­ecular structure of **1** showing the labeling scheme. Displacement ellipsoids drawn at the 50% probability level.

Compounds **1**–**3** (Figs. 1 ▸–3 ▸ ▸) exist as enamine tautomers that contain nearly planar amino-methyl­ene-pentane-2,4-dione cores. The largest deviation from the least-squares plane through the core atoms C1–C7, O1, O2 and N is found for C1 in compound **2** with 0.3252 (9) Å. The enamine double bonds C3=C6 vary from 1.399 (4) to 1.407 (2) Å and the enamine C6=N bonds are in the range 1.308 (2) to 1.321 (4) Å (Tables 1 ▸–3 ▸ ▸). These values are close to those found in the parent compound amino-methyl­ene-pentane-2,4-dione (1.397 and 1.304 Å, respectively; Gróf *et al.*, 2006*a* ▸) and in the related NMe derivative (1.405 and 1.309 Å, respectively; Gróf *et al.*, 2006*b* ▸). The same holds for the corresponding Schiff bases derived from isomeric *o*-, *m*- and *p*-amino­benzoic acids (Halz *et al.*, 2022 ▸), α-amino acids (Hentsch *et al.*, 2014 ▸) or *o*-di­phenyl­phosphinoaniline (Halz *et al.*, 2021 ▸). In summary, these observations clearly indicate that the *R* group attached to the enamine N atom has no significant influence on the bond lengths and angles of the amino-methyl­ene-pentane-2,4-dione moiety. This holds also for the characteristic 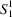 (6) type intra­molecular N—H8⋯O1 hydrogen bonds that change only marginally [*D*⋯*A* distances between 2.597 (4) and 2.6322 (16) Å; Tables 4 ▸–7 ▸ ▸].

Regarding the conformational aspects, 3-[(methyl­amino)­methyl­ene]pentane-2,4-dione may serve as a reference. In this case, the N-bound CH_3_ group exhibits H—C—N—C_enamine_ torsion angles of 1.5 (2), −118.4 (2) and 121.6 (2)° (Gróf *et al.*, 2006*a* ▸). This indicates a nearly ideal *syn*-periplanar orientation of one of the hydrogen atoms and *anti*-periplanar positions for the remaining hydrogen atoms. The formal replacement of one H atom by a phenyl group in the structure of **1** leads to an approximately 26° clockwise rotation of the CH_2_Ph unit along the N—C7 bond. As a result, the hydrogen atoms are now moved to *syn*-periplanar and *anti*-periplanar positions (H9—C7—N—C6 = 27.2°, H10—C7—N—C6 = 144.0°) and the phenyl carbon atom C8 is in an *anti*-clinal position [C8—C7—N—C6 = −94.4 (2)°]. In the case of the ^*t*^Bu derivative **2**, the presence of three equivalent methyl groups leads to a conformation similar to that of the methyl derivative with *syn*-periplanar orientation of one methyl group [C8—C7—N—C6 = −4.03 (14)°] and an *anti*-clinal arrangement for the remaining methyl groups [C9—C7—N—C6 = −134.31 (11)°] C10—C7—N—C6 = 107.27 (11)°]. In the structure of **3**, both the methyl and the phenyl carbon atoms are moved to *anti*-clinal positions [C14—C7—N1—C6 = 115.5 (4)°; 122.4 (3)° for the second mol­ecule], C8—C7—N1—C6 = −119.2 (3)°; 112.8 (3)° for second mol­ecule] and the hydrogen atom resides in a nearly ideal *syn*-periplanar position (H9—C7—N—C6 = −1.47°; 5.16° for the second mol­ecule).

In order to get some insight into how the observed conformations are influenced by crystal packing, the gas phase mol­ecular structures of **1**–**3** were optimized by DFT methods using the *Gaussian 16* program package (Frisch *et al.*, 2016 ▸) at the B3LYP/TZVP/GD3BJ level of theory (Becke, 1993 ▸) with the implemented def2-TZVP basis set (Weigand & Ahlrichs, 2005 ▸) and dispersion correction GD3BJ (Grimme *et al.*, 2011 ▸).

**Figure 4:**
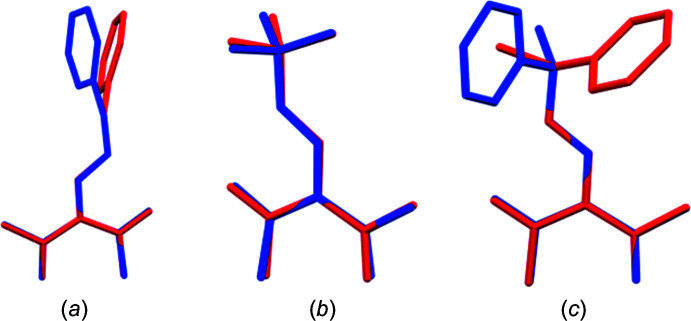
Mol­ecule structure overlay of the experimental (blue) and the calculated structures (red) in **1** (*a*), **2** (*b*) and **3** (*c*), created with *Mercury* (Macrae *et al.*, 2020 ▸).

Bond lengths and angles of the calculated structures are in good agreement with the experimetal data. In Fig. 4 ▸, an overlay of the experimetal (blue) and the calculated structures (red) is shown. Obviously, the planar amino-methyl­ene-pentane-2,4-dione cores fit very well and most of the differences between experimental and theoretical structures are due to the conformations of the organyl groups attached to the enamine nitro­gen atom.

Table 7 ▸ represents a comparison of experimental and calculated torsion angles at the C7—N bond. In the case of compounds **1** and **2**, there is only a moderate increase of the torsion angles with respect to the theoretical values. Additionally, compound **1** exhibits a small change in the orientation of the phenyl group (Fig. 4 ▸ *a*). In the case of compound **3**, the conformational effects are more pronounced and the torsion angles are increased by around 73°. Moreover, the orientation of the phenyl group is also affected (Fig. 4 ▸ *c*).

## 3. Supra­molecular features

**Figure 5:**
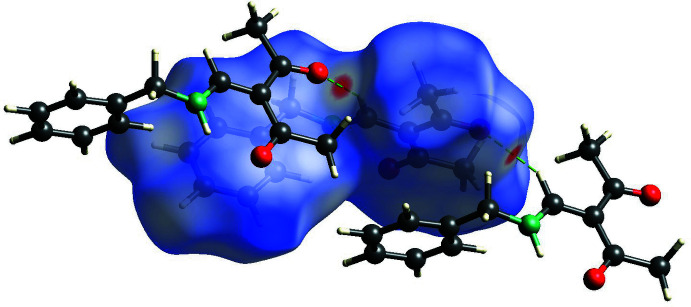
View of the Hirshfeld surface of **1** mapped over *d*_norm_ in the range −0.712 to 0.973 au, showing inter­molecular hydrogen bonds as green dashed lines.

In order to identify the most significant inter­molecular inter­actions, Hirshfeld surface analyses (Spackman *et al.*, 2009 ▸) for compounds **1**–**3** (Figs. 5 ▸–7 ▸ ▸) were carried out with *CrystalExplorer* (Spackman *et al.*, 2021 ▸).

In the case of compound **1** there is a *C*11 (5) type C—H⋯O hydrogen bridge between the enamine CH group (C6—H7) and the acetyl O atom (O2) of of a neighboring mol­ecule (Table 4 ▸, Fig. 8 ▸). This leads to helical chains that propagate in the direction of the *c* axis. Moreover, the packing of the helices is supported by weak π–π inter­actions [3.8747 (12) Å between the centroids of the phenyl groups, 3.79 Å between C3 of the (amino)­methyl­ene-pentane-2,4-dione unit and the centroid of the phenyl ring and 3.42 Å between neighboring (amino)­methyl­ene-pentane-2,4-dione units]. As a result, ribbons extending parallel to [001] are formed, Figs. 9 ▸, 10 ▸.

**Figure 6:**
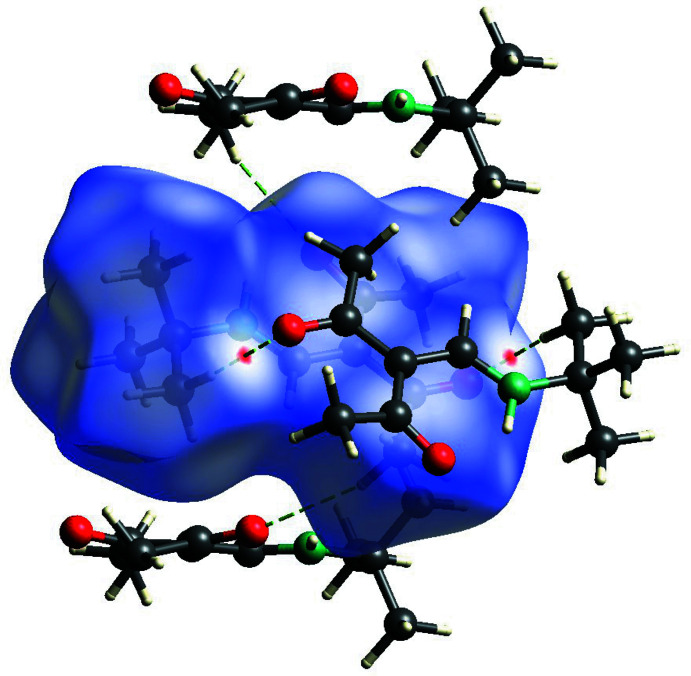
View of the Hirshfeld surface of **2** mapped over *d*_norm_ in the range −0.712 to 0.973 au, showing inter­molecular hydrogen bonds as green dashed lines.

**Figure 8:**
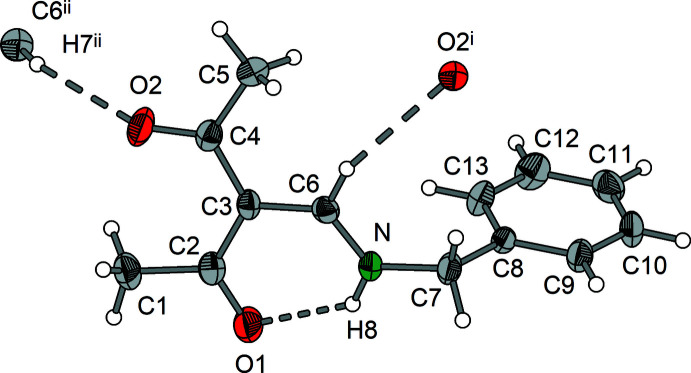
Section of the crystal structure of **1** showing the hydrogen bond. [Symmetry codes: (i) *x*, 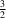 − *y*, − 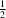 + *z*; (ii) *x*, 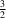 − *y*, 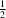 + *z*.]

**Figure 9:**
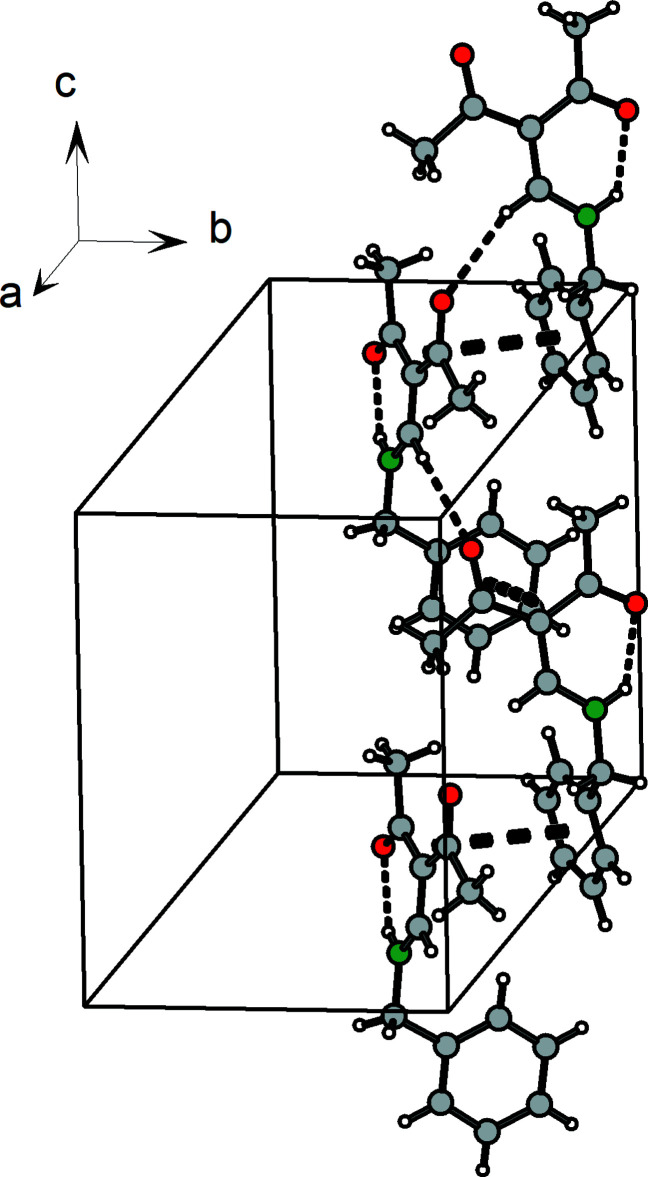
Stacking of mol­ecules in **1** resulting in a layered arrangement parallel to **(**110**)**. Bold dashed lines show the closest contacts with neighboring phenyl and pentane-2,4-dione planes.

**Figure 11:**
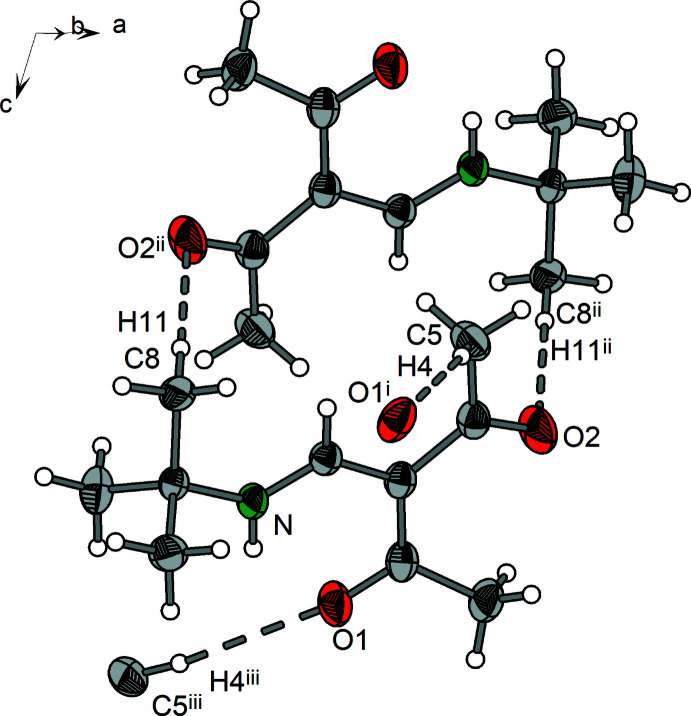
Stacking of the mol­ecules in **2** along the [001] direction.

The Hirshfeld plot of compound **2** reveals that each mol­ecule is involved in four C—H⋯O hydrogen bridges between *t*-butyl groups and acetyl oxygen atoms of neighboring mol­ecules (Fig. 6 ▸). Formally, this can be considered as a formation of dimers based on the complementary hydrogen bridges C8—H11⋯O2′ to give a 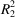 (16) motif. Additionally, the dimers are catenated by 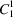 (6) type hydrogen bridges along the *a* axis (Table 5 ▸, Fig. 11 ▸).

**Figure 12:**
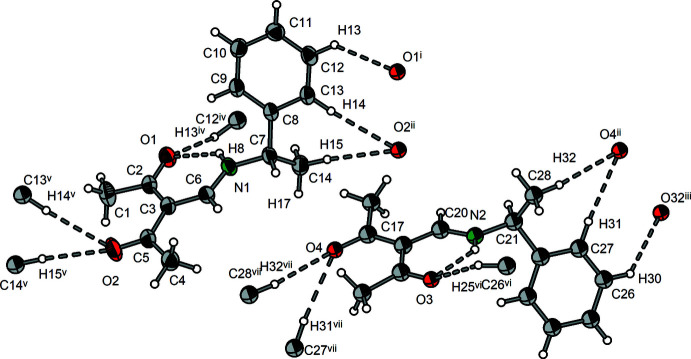
Section of the crystal structure of **3** showing the hydrogen bonds (dashed lines). [Symmetry codes: (i) 1 − *x*, − 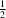 + *y*, −*z*; (ii) 1 − *x*, *y*, *z*; (iii) −*x*, 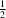 + *y*, 1 − *z*; (iv) 1 − *x*, 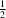 + *y*, −*z*; (v) 1 + *x*, *y*, *z*; (vi) −*x*, − 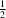 + *y*, 1 − *z*; (vii) 1 + *x*, *y z*.]

**Figure 13:**
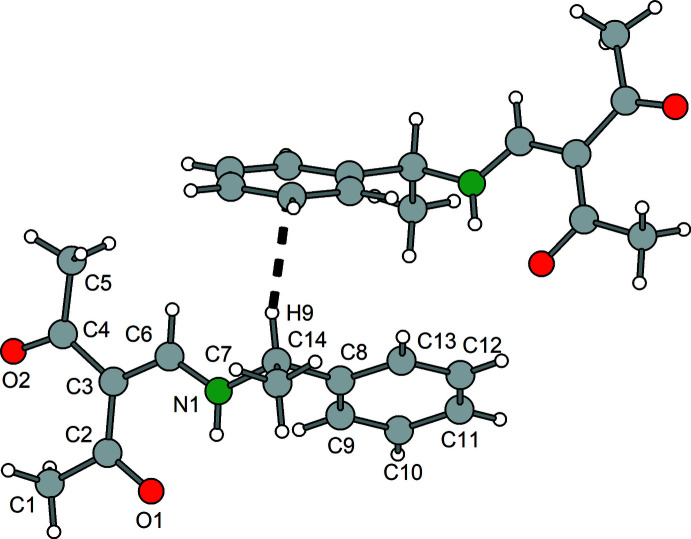
Section of the crystal structure of **3** showing the C—H⋯π inter­action (thick dashed line).

Compound **3** exhibits two major types of inter­actions that are based on C—H⋯O hydrogen bridges (Table 6 ▸) and C—H⋯π contacts with an H⋯*Cg*(phen­yl) distance of 2.68 Å. The C—H⋯O hydrogen bridges are formed between methyl and phenyl groups of the methyl­benzyl residue as donors and acetyl oxygen atoms of neighboring mol­ecules as acceptors (Fig. 12 ▸). In the case of the C—H⋯π inter­action, the benzyl CH fragment and a neighboring phenyl group are involved (Fig. 13 ▸, for mol­ecule 1).

A comparison of the calculated gas phase structures and the experimentally determined structures reveals that the effect of crystal packing is only marginal for compounds **1** and **2**, *i.e.* only minor adjustments of the mol­ecular conformations are required for optimum inter­molecular inter­actions. In contrast to these compounds, **3** requires a stronger mol­ecular reorganization in the solid state and presumably this is in particular due to C—H⋯π inter­actions.

## 4. Database survey

Currently the Cambridge Structural Database (CSD, version 2020.3, Groom *et al.*, 2016) contains 22 entries for Schiff bases with amino-methyl­ene-pentane-2,4-dione cores. In all cases, enamine tautomers are observed.

## 5. Synthesis and crystallization

3-Formyl­acetyl­acetone (3.00 g, 23.4 mmol) and the corresponding amine [1.76 g of benzyl­amine for **1**, 2.57 g of *tert*-butyl­amine for **2** and 2.91 g of (*S*)-methyl-benzyl­amine for **3**, 24.0 mmol] were dissolved in methanol (50 ml) and heated under reflux for one h. After removal of the volatiles *in vacuo*, the residue was washed twice with cold *n*-pentane and afterwards dried *in vacuo*.

Yield: 2.5 g (77%) for **1**, 2.7g (85%) for **2** and 4.3 g (77%) for **3** based on 3-formyl­acetyl­acetone. Compounds **1**–**3** were obtained as yellow air-stable powders that are soluble in polar solvents such as methanol or CHCl_3_ and less soluble in toluene or *n*-hexane.

Crystals suitable for single-crystal X-ray diffraction were obtained by slow evaporation of the solvent from solutions in methanol (compounds **1** and **3**) or diethyl ether (compound **2**).

**Compound 1**: m.p. = 368 K. Elemental Analysis for C_13_H_15_NO_2_: Calculated: C 77.72, H 7.36, N 6.06%. Found: C 77.22, H 7.22, N 5.90%.

IR: 2971 (*m*), 1618 (*vs*), 1570 (*vs*), 1494 (*m*), 1446 (*w*), 1390 (*s*), 1349 (*m*), 1308 (*s*), 1242 (*s*), 1201 (*w*), 1119 (*m*), 1070 (*w*), 1023 (*m*), 975 (*s*), 927 (*m*), 816 (*s*), 755 (*s*), 705 (*s*), 620 (*s*), 552 (*m*), 508 (*m*), 444 (*w*) cm^−1^.

^1^H-NMR (CDCl_3_, 400 MHz) δ = 2.22 (*s*, 3 H, CO—C*H*_3_), 2.47 (*s*, 3 H, CO—C*H_3_*), 4.51 (*d* ^3^ *J* = 5.9 Hz, 2 H, C*H*_2_), 7.22–7.38 (*m*, 5 H, C*H*_aromatic_), 7.77 (*d*, ^3^ *J* = 13,1 Hz, 1 H, C*H*), 11.28 (*s*, 1 H, N*H*) ppm,

^13^C-NMR (CDCl_3_, 100 MHz) δ = 27.2 (–*C*H_3_), 31.8 (–*C*H_3_), 53.7 (–*C*H_2_), 111.9 [C(O)—*C*—C(O)], 127.2 (*C*H_aromatic_), 128.3 (*C*H_aromatic_), 129.0 (*C*H_aromatic_), 135.9 (*C*H_aromatic_), 159.6, (*C*H—NH), 194.2 (*C*O), 200.3 (*C*O) ppm.

**Compound 2**: m.p. = 355 K. Elemental Analysis for C_10_H_17_NO_2_: Calculated: C 65.54, H 9.35, N 7.64%. Found: C 64.78, H 9.26, N 7.33%.

IR: 2970 (*m*), 1649 (*w*), 1608 (*m*), 1578 (*vs*), 1470 (*w*), 1400 (*m*), 1382 (*m*), 1321 (*m*), 1281 (*s*), 1240 (*m*), 1205 (*m*), 1025 (*m*), 990 (*m*), 975 (*s*), 944 (*w*), 928 (*m*), 827 (*s*), 646 (*w*), 626 (*s*), 567 (*s*), 500 (*w*), 474 (*w*), 413 (*w*), 333 (*m*), 305 (*w*), 272 (*w*) cm^−1^.

^1^H-NMR (CDCl_3_, 400 MHz) δ = 1.31 [*s*, 9 H, C(C*H*_3_)], 2.25 (*s*, 3 H, C*H*_3_), 2.45 (*s*, 3 H, C*H*_3_), 7.85 (*d*, ^3^ *J* = 13.7 Hz, 1 H, C*H*), 11.39 (*s*, 1 H, N*H*) ppm.

^13^C-NMR (CDCl_3_, 100 MHz) δ = 27.4 (–*C*H_3_), 29.8 [–C(*C*H_3_)_3_], 31.8 (–*C*H_3_), 53.6 [–*C*(CH_3_)_3_], 111.2 [C(O)—*C–*-C(O)], 155.2, (*C*H—NH), 194.2 (*C*O), 199.8 (*C*O) ppm.

**Compound 3**: m.p. = 335 K. Elemental Analysis for C_14_H_17_NO_2_: Calculated: C 72.70, H 7.41, N 6.06%. Found: C 72.22, H 7.22, N 5.90%.

IR: 2971 (*m*), 1618 (*vs*), 1570 (*vs*), 1494 (*m*), 1446 (*w*), 1390 (*s*), 1349 (*m*), 1308 (*s*), 1242 (*s*), 1201 (*w*), 1119 (*m*), 1070 (*w*), 1023 (*m*), 975 (*s*), 927 (*m*), 816 (*s*), 755 (*s*), 705 (*s*), 620 (*s*), 552 (*m*), 508 (*m*), 444 (*w*) cm^−1^.

^1^H-NMR (CDCl_3_, 400 MHz) δ = 1.62 (*d*, 3 H, NCC*H*_3_), 2.15 (*s*, 3 H,C*H*_3_), 2.47 (*s*, 3 H, (C*H*_3_), 4.56 (*dq*, ^3^ *J* = 6.9 Hz, ^3^ *J* = 7.0 Hz, 1 H, C*H*), 7.25–7.37 (*m*, 5 H, C*H*_aromatic_), 7.72 (*d*, ^3^ *J* = 6,9 Hz, 1 H, C*H*), 11.40 (*s*, 1 H, N*H*) ppm.

^13^C-NMR (CDCl_3_, 100 MHz) δ = 23.4 (NC*C*H_3_), 27.2 (–*C*H_3_),31.9 (–*C*H_3_), 59.1 (N*C*CH_3_), 111.7 [C(O)—*C–*-C(O)], 126.0 (*C*H_aromatic_), 128.2 (*C*H_aromatic_), 129.1 (*C*H_aromatic_), 141.7 (*C*H_aromatic_), 158.2 (*C*H—NH), 194.3 (*C*O,) 200.3 (*C*O) ppm.

## 6. Refinement

Crystal data, data collection and structure refinement details are summarized in Table 8 ▸. Hydrogen atoms were positioned geometrically (C—H = 0.95–0.98 Å) and refined as riding, with *U*_iso_(H) = 1.2*U*_eq_(C) for CH and NH hydrogen atoms and *U*_iso_(H) = 1.5*U*_eq_(C) for CH_3_ hydrogen atoms. The investigated crystal of **3** was twinned by non-merohedry and treated as a two-domain crystal with a refined BASF factor of 0.1151.

## Supplementary Material

Crystal structure: contains datablock(s) 1, 2, 3, global. DOI: 10.1107/S205698902300587X/wm5685sup1.cif

Structure factors: contains datablock(s) 1. DOI: 10.1107/S205698902300587X/wm56851sup5.hkl

Structure factors: contains datablock(s) 2. DOI: 10.1107/S205698902300587X/wm56852sup6.hkl

Structure factors: contains datablock(s) 3. DOI: 10.1107/S205698902300587X/wm56853sup7.hkl

Supporting information file. DOI: 10.1107/S205698902300587X/wm56851sup5.cml

Supporting information file. DOI: 10.1107/S205698902300587X/wm56852sup6.cml

Supporting information file. DOI: 10.1107/S205698902300587X/wm56853sup7.cml

CCDC references: 2279192, 2279191, 2279190

Additional supporting information:  crystallographic information; 3D view; checkCIF report

## Figures and Tables

**Figure 2:**
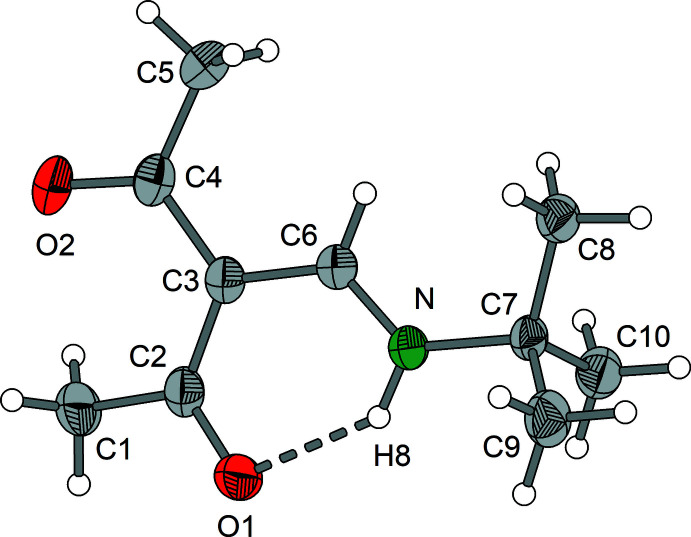
Mol­ecular structure of **2** showing the labeling scheme. Displacement ellipsoids drawn at the 50% probability level.

**Figure 3:**
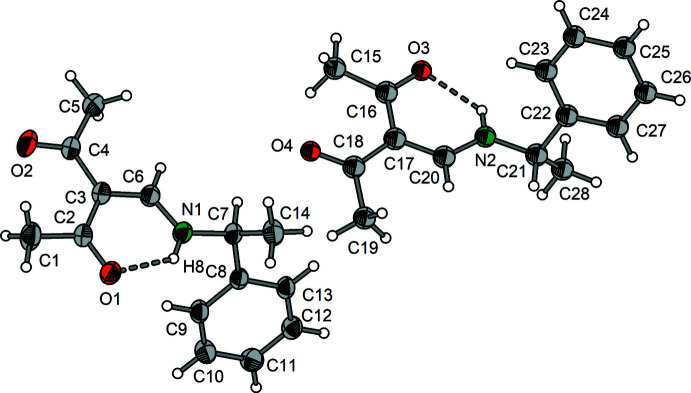
Mol­ecular structure of **3** showing the labeling scheme. Displacement ellipsoids drawn at the 50% probability level.

**Figure 7:**
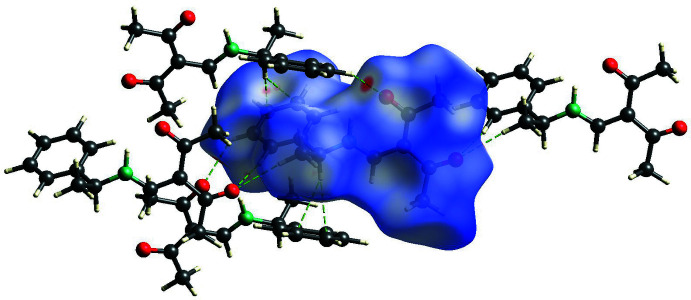
View of the Hirshfeld surface of mol­ecule 1 of compound **3** mapped over *d*_norm_ in the range −0.712 to 0.973 au, showing inter­molecular hydrogen bonds as green dashed lines.

**Figure 10:**
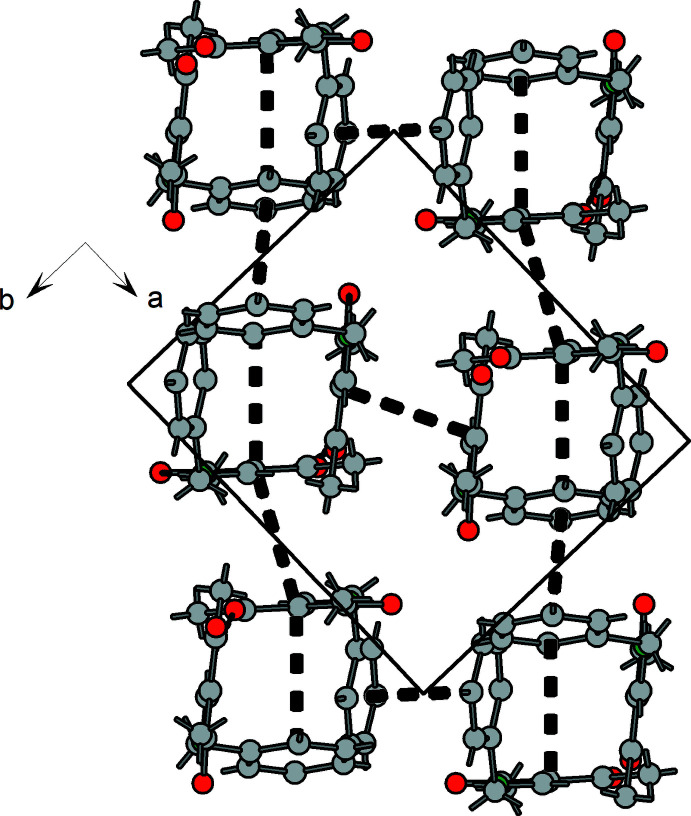
Helical chains of **1** stabilized by hydrogen bonds (thin dashed lines) and π–π inter­actions.

**Table 1:** Selected geometric parameters (Å, °) for **1** [Chem scheme1]

C1—C2	1.511 (2)	C7—N	1.462 (2)
C2—O1	1.245 (2)	C7—C8	1.519 (2)
C2—C3	1.456 (2)	C8—C13	1.384 (2)
C3—C6	1.404 (2)	C8—C9	1.393 (2)
C3—C4	1.469 (2)	C9—C10	1.384 (2)
C4—O2	1.2350 (19)	C10—C11	1.379 (3)
C4—C5	1.512 (3)	C11—C12	1.388 (3)
C6—N	1.316 (2)	C12—C13	1.386 (2)
			
C8—C7—N—C6	−94.4 (2)		

**Table 2:** Selected geometric parameters (Å, °) for **2** [Chem scheme1]

C1—C2	1.5042 (13)	C4—O2	1.2287 (12)
C2—O1	1.2419 (12)	C4—C5	1.5135 (15)
C2—C3	1.4502 (14)	C7—C8	1.5197 (14)
C3—C6	1.4043 (12)	C7—C9	1.5235 (14)
C3—C4	1.4578 (13)	C7—C10	1.5237 (14)
			
C8—C7—N—C6	−14.03 (14)	C10—C7—N—C6	107.27 (11)
C9—C7—N—C6	−134.31 (11)		

**Table 3:** Selected geometric parameters (Å, °) for **3** [Chem scheme1]

C1—C2	1.504 (5)	C15—C16	1.510 (5)
C2—O1	1.246 (4)	C16—O3	1.236 (4)
C2—C3	1.453 (4)	C16—C17	1.455 (4)
C3—C6	1.399 (4)	C17—C20	1.396 (4)
C3—C4	1.472 (5)	C17—C18	1.468 (4)
C4—O2	1.224 (4)	C18—O4	1.228 (4)
C4—C5	1.517 (5)	C18—C19	1.518 (5)
C6—N1	1.311 (4)	C20—N2	1.321 (4)
C7—N1	1.470 (4)	C21—N2	1.463 (4)
C7—C8	1.522 (4)	C21—C28	1.524 (5)
C7—C14	1.527 (5)	C21—C22	1.534 (4)
			
C8—C7—N1—C6	−119.2 (3)	C22—C21—N2—C20	−112.8 (3)
C14—C7—N1—C6	115.5 (4)	C28—C21—N2—C20	122.4 (3)

**Table 4:** Hydrogen-bond geometry (Å, °) for **1** [Chem scheme1]

*D*—H⋯*A*	*D*—H	H⋯*A*	*D*⋯*A*	*D*—H⋯*A*
N—H8⋯O1	0.87	1.97	2.6177 (18)	130
C6—H7⋯O2^i^	0.94	2.57	3.434 (2)	154

Symmetry code: (i) 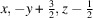 .

**Table 5:** Hydrogen-bond geometry (Å, °) for **2** [Chem scheme1]

*D*—H⋯*A*	*D*—H	H⋯*A*	*D*⋯*A*	*D*—H⋯*A*
N—H8⋯O1	0.90 (2)	1.90 (2)	2.6322 (16)	136 (2)
C5—H4⋯O1^i^	0.97 (2)	2.69 (2)	3.630 (2)	163 (2)
C8—H11⋯O2^ii^	0.98 (2)	2.67 (2)	3.642 (2)	174 (1)

Symmetry codes: (i) 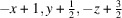 ; (ii) 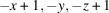 .

**Table 6:** Hydrogen-bond geometry (Å, °) for **3** [Chem scheme1]

*D*—H⋯*A*	*D*—H	H⋯*A*	*D*⋯*A*	*D*—H⋯*A*
N1—H8⋯O1	0.88	1.94	2.597 (4)	131
N2—H25⋯O3	0.88	1.95	2.603 (4)	130
C14—H15⋯O2^i^	0.98	2.55	3.532 (5)	175
C14—H17⋯O4	0.98	2.66	3.482 (5)	142
C28—H32⋯O4^i^	0.98	2.54	3.512 (5)	173

Symmetry code: (i) 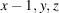 .

**Table 7:** Comparison of torsion angles in the crystal structures of **1**–**3** and from theoretical DFT calculations

Compound	Torsion angle	Crystal structure determination	DFT calculation
**1**	C8—C7—N—C6	–94.4 (1)	–107.8
**2**	C8—C7—N—C6	−14.03 (14)	0.0
	C9—C7—N—C6	–134.31 (11)	–120.8
	C10—C7—N—C6	107.27 (11)	120.8
**3**	C8—C7—N1—C6	–119.2 (4); −112.8 (3)*	–43.3
	C14—C7—N1—C6	115.5 (4); 122.4 (3)*	–167.3

*Values for the comparable bond in the second mol­ecule.

**Table 8:** Experimental details

	**1**	**2**	**3**
Crystal data			
Chemical formula	C_13_H_15_NO_2_	C_10_H_17_NO_2_	C_14_H_17_NO_2_
*M*_r_	217.26	183.24	231.28
Crystal system, space group	Monoclinic, *P*2_1_/*c*	Monoclinic, *P*2_1_/*c*	Monoclinic, *P*2_1_
Temperature (K)	213	170	170
*a*, *b*, *c* (Å)	11.7356 (14), 9.2401 (8), 11.3970 (14)	9.8226 (7), 9.8323 (6), 11.2700 (8)	10.0459 (9), 8.1011 (5), 15.7052 (13)
β (°)	113.148 (14)	108.171 (5)	103.372 (7)
*V* (Å^3^)	1136.4 (2)	1034.16 (12)	1243.48 (17)
*Z*	4	4	4
Radiation type	Mo *K*α	Mo *K*α	Mo *K*α
μ (mm^−1^)	0.09	0.08	0.08
Crystal size (mm)	0.33 × 0.15 × 0.12	0.32 × 0.28 × 0.21	0.44 × 0.21 × 0.14

Data collection			
Diffractometer	Stoe IPDS 2	Stoe IPDS 2	Stoe IPDS 2T
No. of measured, independent and observed [*I* > 2σ(*I*)] reflections	7396, 2159, 1414	7337, 2774, 2126	11343, 11343, 9181
*R*_int_	0.078	0.033	0.042
(sin θ/λ)_max_ (Å^−1^)	0.616	0.685	0.688

Refinement			
*R*[*F* ^2^ > 2σ(*F* ^2^)], *wR*(*F* ^2^), *S*	0.041, 0.097, 0.94	0.036, 0.104, 1.03	0.048, 0.131, 1.05
No. of reflections	2159	2774	11343
No. of parameters	147	123	315
No. of restraints	0	0	1
H-atom treatment	H-atom parameters constrained	H-atom parameters constrained	H-atom parameters constrained
Δρ_max_, Δρ_min_ (e Å^−3^)	0.18, −0.17	0.30, −0.14	0.27, −0.28
Absolute structure	–	–	Classical Flack method preferred over Parsons because s.u. lower.
Absolute structure parameter	–	–	2.9 (7)

Computer programs: *X-AREA WinXpose* (Stoe, 2016 ▸), *X-AREA Recipe* (Stoe, 2015 ▸), *X-AREA* (Stoe, 2016 ▸), *SHELXT* (Sheldrick, 2015*a* ▸), *SHELXL* (Sheldrick, 2015*b* ▸), *DIAMOND* (Brandenburg, 2019 ▸) and *OLEX2* (Dolomanov *et al.*, 2009 ▸).

## supplementary crystallographic information

### 3-[(Benzylamino)methylidene]pentane-2,4-dione (1) Crystal data

**Table:**

C_13_H_15_NO_2_	*F*(000) = 464
*M**_r_* = 217.26	*D*_x_ = 1.270 Mg m^−^^3^
Monoclinic, *P*2_1_/*c*	Mo *K*α radiation, λ = 0.71073 Å
*a* = 11.7356 (14) Å	Cell parameters from 7396 reflections
*b* = 9.2401 (8) Å	θ = 2.9–26.0°
*c* = 11.3970 (14) Å	µ = 0.09 mm^−^^1^
β = 113.148 (14)°	*T* = 213 K
*V* = 1136.4 (2) Å^3^	Block, clear yellow
*Z* = 4	0.33 × 0.15 × 0.12 mm

### 3-[(Benzylamino)methylidene]pentane-2,4-dione (1) Data collection

**Table:**

STOE IPDS 2 diffractometer	*R*_int_ = 0.078
rotation scans	θ_max_ = 26.0°, θ_min_ = 2.9°
7396 measured reflections	*h* = −13→13
2159 independent reflections	*k* = −11→11
1414 reflections with *I* > 2σ(*I*)	*l* = −13→13

### 3-[(Benzylamino)methylidene]pentane-2,4-dione (1) Refinement

**Table:**

Refinement on *F*^2^	Primary atom site location: iterative
Least-squares matrix: full	Hydrogen site location: inferred from neighbouring sites
*R*[*F*^2^ > 2σ(*F*^2^)] = 0.041	H-atom parameters constrained
*wR*(*F*^2^) = 0.097	*w* = 1/[σ^2^(*F*_o_^2^) + (0.0469*P*)^2^] where *P* = (*F*_o_^2^ + 2*F*_c_^2^)/3
*S* = 0.94	(Δ/σ)_max_ < 0.001
2159 reflections	Δρ_max_ = 0.18 e Å^−^^3^
147 parameters	Δρ_min_ = −0.17 e Å^−^^3^
0 restraints	

### 3-[(Benzylamino)methylidene]pentane-2,4-dione (1) Special details

**Table:**

Geometry. All esds (except the esd in the dihedral angle between two l.s. planes) are estimated using the full covariance matrix. The cell esds are taken into account individually in the estimation of esds in distances, angles and torsion angles; correlations between esds in cell parameters are only used when they are defined by crystal symmetry. An approximate (isotropic) treatment of cell esds is used for estimating esds involving l.s. planes.

### 3-[(Benzylamino)methylidene]pentane-2,4-dione (1) Fractional atomic coordinates and isotropic or equivalent isotropic displacement parameters (Å^2^)

**Table:**

	*x*	*y*	*z*	*U*_iso_*/*U*_eq_	
C1	0.29032 (19)	0.4837 (2)	1.16212 (15)	0.0391 (5)	
H1	0.2334	0.4136	1.1724	0.059*	
H2	0.2718	0.5790	1.1858	0.059*	
H3	0.3747	0.4574	1.2165	0.059*	
C2	0.27629 (16)	0.48567 (19)	1.02452 (14)	0.0284 (4)	
C3	0.34838 (15)	0.58355 (17)	0.98050 (14)	0.0239 (4)	
C4	0.43034 (16)	0.69625 (18)	1.06114 (14)	0.0265 (4)	
C5	0.4992 (2)	0.7943 (2)	1.00520 (17)	0.0404 (5)	
H4	0.4405	0.8413	0.9294	0.061*	
H5	0.5571	0.7374	0.9827	0.061*	
H6	0.5442	0.8670	1.0676	0.061*	
C6	0.34363 (15)	0.56658 (17)	0.85623 (14)	0.0244 (4)	
H7	0.3949	0.6277	0.8324	0.029*	
C7	0.27877 (17)	0.45955 (19)	0.64354 (14)	0.0297 (4)	
H9	0.2677	0.3573	0.6186	0.036*	
H10	0.3610	0.4890	0.6492	0.036*	
C8	0.18161 (16)	0.54814 (17)	0.53978 (14)	0.0235 (4)	
C9	0.15894 (17)	0.51739 (19)	0.41290 (15)	0.0307 (4)	
H11	0.2017	0.4412	0.3935	0.037*	
C10	0.07407 (18)	0.5979 (2)	0.31512 (15)	0.0353 (5)	
H12	0.0588	0.5754	0.2298	0.042*	
C11	0.01179 (18)	0.7110 (2)	0.34239 (16)	0.0363 (5)	
H13	−0.0444	0.7670	0.2760	0.044*	
C12	0.03245 (19)	0.7418 (2)	0.46829 (17)	0.0394 (5)	
H14	−0.0108	0.8178	0.4873	0.047*	
C13	0.11704 (17)	0.66035 (19)	0.56594 (16)	0.0329 (4)	
H15	0.1307	0.6816	0.6511	0.040*	
N	0.27486 (14)	0.47394 (15)	0.76962 (11)	0.0290 (4)	
H8	0.2245	0.4184	0.7884	0.035*	
O1	0.20370 (13)	0.39610 (15)	0.95112 (11)	0.0439 (4)	
O2	0.44589 (13)	0.71536 (15)	1.17367 (10)	0.0418 (4)	

### 3-[(Benzylamino)methylidene]pentane-2,4-dione (1) Atomic displacement parameters (Å^2^)

**Table:**

	*U*^11^	*U*^22^	*U*^33^	*U*^12^	*U*^13^	*U*^23^
C1	0.0467 (13)	0.0475 (12)	0.0277 (9)	−0.0032 (10)	0.0196 (9)	0.0032 (8)
C2	0.0266 (10)	0.0345 (10)	0.0231 (8)	0.0034 (8)	0.0087 (7)	0.0020 (7)
C3	0.0247 (10)	0.0277 (9)	0.0173 (7)	0.0044 (7)	0.0063 (7)	0.0020 (6)
C4	0.0271 (10)	0.0277 (9)	0.0220 (8)	0.0066 (7)	0.0068 (7)	−0.0002 (7)
C5	0.0527 (14)	0.0324 (10)	0.0329 (9)	−0.0132 (9)	0.0135 (9)	−0.0055 (8)
C6	0.0246 (10)	0.0226 (8)	0.0234 (8)	0.0020 (7)	0.0066 (7)	0.0039 (7)
C7	0.0372 (11)	0.0319 (9)	0.0194 (8)	0.0020 (8)	0.0104 (7)	−0.0029 (7)
C8	0.0253 (10)	0.0253 (8)	0.0197 (8)	−0.0045 (7)	0.0087 (7)	−0.0024 (6)
C9	0.0364 (11)	0.0335 (10)	0.0230 (8)	0.0026 (8)	0.0125 (7)	−0.0022 (7)
C10	0.0423 (12)	0.0435 (11)	0.0186 (8)	−0.0020 (9)	0.0105 (8)	0.0006 (8)
C11	0.0345 (12)	0.0358 (11)	0.0311 (9)	0.0005 (8)	0.0049 (8)	0.0089 (8)
C12	0.0378 (12)	0.0376 (11)	0.0389 (10)	0.0106 (9)	0.0107 (9)	−0.0035 (8)
C13	0.0345 (11)	0.0383 (10)	0.0239 (8)	0.0019 (8)	0.0092 (8)	−0.0076 (7)
N	0.0351 (9)	0.0317 (8)	0.0184 (7)	−0.0059 (7)	0.0087 (6)	−0.0007 (6)
O1	0.0468 (9)	0.0559 (9)	0.0285 (7)	−0.0222 (7)	0.0145 (6)	−0.0036 (6)
O2	0.0464 (9)	0.0535 (8)	0.0232 (6)	−0.0082 (7)	0.0111 (6)	−0.0115 (6)

### 3-[(Benzylamino)methylidene]pentane-2,4-dione (1) Geometric parameters (Å, º)

**Table:**

C1—C2	1.511 (2)	C7—C8	1.519 (2)
C1—H1	0.9700	C7—H9	0.9800
C1—H2	0.9700	C7—H10	0.9800
C1—H3	0.9700	C8—C13	1.384 (2)
C2—O1	1.245 (2)	C8—C9	1.393 (2)
C2—C3	1.456 (2)	C9—C10	1.384 (2)
C3—C6	1.404 (2)	C9—H11	0.9400
C3—C4	1.469 (2)	C10—C11	1.379 (3)
C4—O2	1.2350 (19)	C10—H12	0.9400
C4—C5	1.512 (3)	C11—C12	1.388 (3)
C5—H4	0.9700	C11—H13	0.9400
C5—H5	0.9700	C12—C13	1.386 (2)
C5—H6	0.9700	C12—H14	0.9400
C6—N	1.316 (2)	C13—H15	0.9400
C6—H7	0.9400	N—H8	0.8700
C7—N	1.462 (2)		
			
C2—C1—H1	109.5	C8—C7—H9	108.7
C2—C1—H2	109.5	N—C7—H10	108.7
H1—C1—H2	109.5	C8—C7—H10	108.7
C2—C1—H3	109.5	H9—C7—H10	107.6
H1—C1—H3	109.5	C13—C8—C9	118.63 (16)
H2—C1—H3	109.5	C13—C8—C7	122.88 (14)
O1—C2—C3	121.22 (14)	C9—C8—C7	118.47 (15)
O1—C2—C1	117.12 (16)	C10—C9—C8	120.64 (17)
C3—C2—C1	121.60 (15)	C10—C9—H11	119.7
C6—C3—C2	118.70 (15)	C8—C9—H11	119.7
C6—C3—C4	117.82 (15)	C11—C10—C9	120.23 (16)
C2—C3—C4	123.44 (14)	C11—C10—H12	119.9
O2—C4—C3	122.95 (16)	C9—C10—H12	119.9
O2—C4—C5	117.85 (16)	C10—C11—C12	119.73 (17)
C3—C4—C5	119.20 (14)	C10—C11—H13	120.1
C4—C5—H4	109.5	C12—C11—H13	120.1
C4—C5—H5	109.5	C13—C12—C11	119.84 (18)
H4—C5—H5	109.5	C13—C12—H14	120.1
C4—C5—H6	109.5	C11—C12—H14	120.1
H4—C5—H6	109.5	C8—C13—C12	120.91 (16)
H5—C5—H6	109.5	C8—C13—H15	119.5
N—C6—C3	126.77 (16)	C12—C13—H15	119.5
N—C6—H7	116.6	C6—N—C7	123.33 (16)
C3—C6—H7	116.6	C6—N—H8	118.3
N—C7—C8	114.34 (15)	C7—N—H8	118.3
N—C7—H9	108.7		
			
O1—C2—C3—C6	−5.9 (2)	N—C7—C8—C9	−165.58 (16)
C1—C2—C3—C6	171.41 (17)	C13—C8—C9—C10	0.3 (3)
O1—C2—C3—C4	176.69 (17)	C7—C8—C9—C10	−178.03 (17)
C1—C2—C3—C4	−6.0 (3)	C8—C9—C10—C11	0.8 (3)
C6—C3—C4—O2	−176.37 (17)	C9—C10—C11—C12	−1.5 (3)
C2—C3—C4—O2	1.1 (3)	C10—C11—C12—C13	1.1 (3)
C6—C3—C4—C5	3.8 (2)	C9—C8—C13—C12	−0.7 (3)
C2—C3—C4—C5	−178.77 (16)	C7—C8—C13—C12	177.57 (18)
C2—C3—C6—N	3.6 (3)	C11—C12—C13—C8	0.0 (3)
C4—C3—C6—N	−178.82 (15)	C3—C6—N—C7	−177.67 (16)
N—C7—C8—C13	16.1 (2)	C8—C7—N—C6	−94.4 (2)

### 3-[(Benzylamino)methylidene]pentane-2,4-dione (1) Hydrogen-bond geometry (Å, º)

**Table:**

*D*—H···*A*	*D*—H	H···*A*	*D*···*A*	*D*—H···*A*
N—H8···O1	0.87	1.97	2.6177 (18)	130
C6—H7···O2^i^	0.94	2.57	3.434 (2)	154

Symmetry code: (i) *x*, −*y*+3/2, *z*−1/2.

### 3-[(tert-Butylamino)methylidene]pentan-2,4-dione (2) Crystal data

**Table:**

C_10_H_17_NO_2_	*F*(000) = 400
*M**_r_* = 183.24	*D*_x_ = 1.177 Mg m^−^^3^
Monoclinic, *P*2_1_/*c*	Mo *K*α radiation, λ = 0.71073 Å
*a* = 9.8226 (7) Å	Cell parameters from 2011 reflections
*b* = 9.8323 (6) Å	θ = 3.7–29.5°
*c* = 11.2700 (8) Å	µ = 0.08 mm^−^^1^
β = 108.171 (5)°	*T* = 170 K
*V* = 1034.16 (12) Å^3^	Block, colourless
*Z* = 4	0.32 × 0.28 × 0.21 mm

### 3-[(tert-Butylamino)methylidene]pentan-2,4-dione (2) Data collection

**Table:**

STOE IPDS 2 diffractometer	*R*_int_ = 0.033
rotation scans	θ_max_ = 29.1°, θ_min_ = 2.8°
7337 measured reflections	*h* = −13→13
2774 independent reflections	*k* = −13→13
2126 reflections with *I* > 2σ(*I*)	*l* = −11→15

### 3-[(tert-Butylamino)methylidene]pentan-2,4-dione (2) Refinement

**Table:**

Refinement on *F*^2^	Primary atom site location: iterative
Least-squares matrix: full	Hydrogen site location: inferred from neighbouring sites
*R*[*F*^2^ > 2σ(*F*^2^)] = 0.036	H-atom parameters constrained
*wR*(*F*^2^) = 0.104	*w* = 1/[σ^2^(*F*_o_^2^) + (0.0562*P*)^2^ + 0.1178*P*] where *P* = (*F*_o_^2^ + 2*F*_c_^2^)/3
*S* = 1.03	(Δ/σ)_max_ < 0.001
2774 reflections	Δρ_max_ = 0.30 e Å^−^^3^
123 parameters	Δρ_min_ = −0.14 e Å^−^^3^
0 restraints	

### 3-[(tert-Butylamino)methylidene]pentan-2,4-dione (2) Special details

**Table:**

Geometry. All esds (except the esd in the dihedral angle between two l.s. planes) are estimated using the full covariance matrix. The cell esds are taken into account individually in the estimation of esds in distances, angles and torsion angles; correlations between esds in cell parameters are only used when they are defined by crystal symmetry. An approximate (isotropic) treatment of cell esds is used for estimating esds involving l.s. planes.

### 3-[(tert-Butylamino)methylidene]pentan-2,4-dione (2) Fractional atomic coordinates and isotropic or equivalent isotropic displacement parameters (Å^2^)

**Table:**

	*x*	*y*	*z*	*U*_iso_*/*U*_eq_	
C1	0.83575 (10)	0.46662 (12)	0.88153 (11)	0.0344 (2)	
H1	0.8632	0.4892	0.9706	0.052*	
H2	0.8823	0.5299	0.8392	0.052*	
H3	0.8660	0.3735	0.8715	0.052*	
C2	0.67575 (9)	0.47771 (10)	0.82522 (10)	0.0268 (2)	
C3	0.60104 (9)	0.41294 (10)	0.70726 (9)	0.0247 (2)	
C4	0.67422 (10)	0.36658 (10)	0.62027 (10)	0.0274 (2)	
C5	0.59161 (12)	0.28835 (12)	0.50456 (10)	0.0351 (2)	
H4	0.5154	0.3460	0.4516	0.053*	
H5	0.5493	0.2070	0.5290	0.053*	
H6	0.6567	0.2614	0.4582	0.053*	
C6	0.45217 (10)	0.39420 (10)	0.67456 (9)	0.0245 (2)	
H7	0.4075	0.3445	0.6003	0.029*	
C7	0.21415 (9)	0.41512 (10)	0.71095 (9)	0.0242 (2)	
C8	0.14674 (10)	0.35776 (12)	0.58053 (9)	0.0305 (2)	
H9	0.1901	0.2694	0.5740	0.046*	
H10	0.1632	0.4206	0.5189	0.046*	
H11	0.0435	0.3463	0.5647	0.046*	
C9	0.14777 (11)	0.55234 (11)	0.72323 (12)	0.0355 (2)	
H12	0.0449	0.5409	0.7097	0.053*	
H13	0.1622	0.6154	0.6609	0.053*	
H14	0.1935	0.5891	0.8071	0.053*	
C10	0.19622 (11)	0.31624 (12)	0.80911 (10)	0.0324 (2)	
H15	0.0940	0.3027	0.7973	0.049*	
H16	0.2428	0.3535	0.8926	0.049*	
H17	0.2403	0.2289	0.8006	0.049*	
N	0.36968 (8)	0.43920 (9)	0.73734 (8)	0.02652 (19)	
H8	0.4115	0.4898	0.8030	0.032*	
O1	0.60963 (7)	0.53863 (9)	0.88675 (8)	0.0377 (2)	
O2	0.80125 (8)	0.39082 (10)	0.63547 (8)	0.0414 (2)	

### 3-[(tert-Butylamino)methylidene]pentan-2,4-dione (2) Atomic displacement parameters (Å^2^)

**Table:**

	*U*^11^	*U*^22^	*U*^33^	*U*^12^	*U*^13^	*U*^23^
C1	0.0207 (4)	0.0420 (6)	0.0379 (6)	−0.0005 (4)	0.0056 (4)	−0.0040 (5)
C2	0.0207 (4)	0.0270 (5)	0.0328 (5)	−0.0015 (3)	0.0084 (4)	−0.0005 (4)
C3	0.0202 (4)	0.0253 (4)	0.0296 (5)	−0.0003 (3)	0.0092 (3)	0.0014 (4)
C4	0.0250 (4)	0.0272 (5)	0.0326 (5)	0.0005 (4)	0.0126 (4)	0.0039 (4)
C5	0.0353 (5)	0.0404 (6)	0.0342 (6)	−0.0024 (4)	0.0174 (4)	−0.0044 (4)
C6	0.0217 (4)	0.0265 (4)	0.0255 (4)	−0.0005 (3)	0.0078 (3)	0.0008 (4)
C7	0.0172 (4)	0.0296 (4)	0.0259 (5)	−0.0008 (3)	0.0068 (3)	−0.0009 (4)
C8	0.0230 (4)	0.0421 (6)	0.0250 (5)	−0.0027 (4)	0.0056 (4)	−0.0015 (4)
C9	0.0263 (5)	0.0335 (5)	0.0477 (7)	0.0026 (4)	0.0131 (4)	−0.0034 (5)
C10	0.0310 (5)	0.0387 (6)	0.0288 (5)	−0.0018 (4)	0.0109 (4)	0.0020 (4)
N	0.0177 (3)	0.0336 (4)	0.0279 (4)	−0.0015 (3)	0.0065 (3)	−0.0055 (3)
O1	0.0239 (3)	0.0462 (5)	0.0420 (5)	−0.0017 (3)	0.0089 (3)	−0.0172 (4)
O2	0.0270 (4)	0.0555 (5)	0.0479 (5)	−0.0069 (3)	0.0207 (3)	−0.0060 (4)

### 3-[(tert-Butylamino)methylidene]pentan-2,4-dione (2) Geometric parameters (Å, º)

**Table:**

C1—C2	1.5042 (13)	C7—N	1.4815 (11)
C1—H1	0.9800	C7—C8	1.5197 (14)
C1—H2	0.9800	C7—C9	1.5235 (14)
C1—H3	0.9800	C7—C10	1.5237 (14)
C2—O1	1.2419 (12)	C8—H9	0.9800
C2—C3	1.4502 (14)	C8—H10	0.9800
C3—C6	1.4043 (12)	C8—H11	0.9800
C3—C4	1.4578 (13)	C9—H12	0.9800
C4—O2	1.2287 (12)	C9—H13	0.9800
C4—C5	1.5135 (15)	C9—H14	0.9800
C5—H4	0.9800	C10—H15	0.9800
C5—H5	0.9800	C10—H16	0.9800
C5—H6	0.9800	C10—H17	0.9800
C6—N	1.3079 (12)	N—H8	0.8800
C6—H7	0.9500		
			
C2—C1—H1	109.5	C8—C7—C9	110.29 (8)
C2—C1—H2	109.5	N—C7—C10	107.56 (8)
H1—C1—H2	109.5	C8—C7—C10	110.56 (8)
C2—C1—H3	109.5	C9—C7—C10	110.45 (8)
H1—C1—H3	109.5	C7—C8—H9	109.5
H2—C1—H3	109.5	C7—C8—H10	109.5
O1—C2—C3	121.36 (8)	H9—C8—H10	109.5
O1—C2—C1	117.32 (9)	C7—C8—H11	109.5
C3—C2—C1	121.22 (8)	H9—C8—H11	109.5
C6—C3—C2	119.08 (8)	H10—C8—H11	109.5
C6—C3—C4	118.29 (9)	C7—C9—H12	109.5
C2—C3—C4	122.63 (8)	C7—C9—H13	109.5
O2—C4—C3	122.54 (10)	H12—C9—H13	109.5
O2—C4—C5	117.84 (9)	C7—C9—H14	109.5
C3—C4—C5	119.61 (8)	H12—C9—H14	109.5
C4—C5—H4	109.5	H13—C9—H14	109.5
C4—C5—H5	109.5	C7—C10—H15	109.5
H4—C5—H5	109.5	C7—C10—H16	109.5
C4—C5—H6	109.5	H15—C10—H16	109.5
H4—C5—H6	109.5	C7—C10—H17	109.5
H5—C5—H6	109.5	H15—C10—H17	109.5
N—C6—C3	125.72 (9)	H16—C10—H17	109.5
N—C6—H7	117.1	C6—N—C7	127.69 (8)
C3—C6—H7	117.1	C6—N—H8	116.2
N—C7—C8	111.38 (8)	C7—N—H8	116.2
N—C7—C9	106.50 (8)		
			
O1—C2—C3—C6	−13.30 (15)	C2—C3—C4—C5	174.41 (9)
C1—C2—C3—C6	163.02 (9)	C2—C3—C6—N	5.34 (15)
O1—C2—C3—C4	166.44 (10)	C4—C3—C6—N	−174.40 (9)
C1—C2—C3—C4	−17.25 (15)	C3—C6—N—C7	−175.98 (9)
C6—C3—C4—O2	172.63 (10)	C8—C7—N—C6	−14.03 (14)
C2—C3—C4—O2	−7.11 (15)	C9—C7—N—C6	−134.31 (11)
C6—C3—C4—C5	−5.85 (14)	C10—C7—N—C6	107.27 (11)

### 3-[(tert-Butylamino)methylidene]pentan-2,4-dione (2) Hydrogen-bond geometry (Å, º)

**Table:**

*D*—H···*A*	*D*—H	H···*A*	*D*···*A*	*D*—H···*A*
N—H8···O1	0.90 (2)	1.90 (2)	2.6322 (16)	136 (2)
C5—H4···O1^i^	0.97 (2)	2.69 (2)	3.630 (2)	163 (2)
C8—H11···O2^ii^	0.98 (2)	2.67 (2)	3.642 (2)	174 (1)

Symmetry codes: (i) −*x*+1, *y*+1/2, −*z*+3/2; (ii) −*x*+1, −*y*, −*z*+1.

### 3-{[(S)-Benzyl(methyl)amino]methylidene}pentane-2,4-dione (3) Crystal data

**Table:**

C_14_H_17_NO_2_	*F*(000) = 496
*M**_r_* = 231.28	*D*_x_ = 1.235 Mg m^−^^3^
Monoclinic, *P*2_1_	Mo *K*α radiation, λ = 0.71073 Å
*a* = 10.0459 (9) Å	Cell parameters from 17500 reflections
*b* = 8.1011 (5) Å	θ = 2.5–29.7°
*c* = 15.7052 (13) Å	µ = 0.08 mm^−^^1^
β = 103.372 (7)°	*T* = 170 K
*V* = 1243.48 (17) Å^3^	Block, colourless
*Z* = 4	0.44 × 0.21 × 0.14 mm

### 3-{[(S)-Benzyl(methyl)amino]methylidene}pentane-2,4-dione (3) Data collection

**Table:**

STOE IPDS 2T diffractometer	*R*_int_ = 0.042
rotation scans	θ_max_ = 29.3°, θ_min_ = 2.7°
11343 measured reflections	*h* = −13→13
11343 independent reflections	*k* = −11→11
9181 reflections with *I* > 2σ(*I*)	*l* = −21→21

### 3-{[(S)-Benzyl(methyl)amino]methylidene}pentane-2,4-dione (3) Refinement

**Table:**

Refinement on *F*^2^	H-atom parameters constrained
Least-squares matrix: full	*w* = 1/[σ^2^(*F*_o_^2^) + (0.059*P*)^2^ + 0.3675*P*] where *P* = (*F*_o_^2^ + 2*F*_c_^2^)/3
*R*[*F*^2^ > 2σ(*F*^2^)] = 0.048	(Δ/σ)_max_ < 0.001
*wR*(*F*^2^) = 0.131	Δρ_max_ = 0.27 e Å^−^^3^
*S* = 1.05	Δρ_min_ = −0.28 e Å^−^^3^
11343 reflections	Extinction correction: SHELXL-2018/3 (Sheldrick 2014, Fc^*^=kFc[1+0.001xFc^2^λ^3^/sin(2θ)]^-1/4^
315 parameters	Extinction coefficient: 0.044 (6)
1 restraint	Absolute structure: Classical Flack method preferred over Parsons because s.u. lower.
Primary atom site location: iterative	Absolute structure parameter: 2.9 (7)
Hydrogen site location: inferred from neighbouring sites	

### 3-{[(S)-Benzyl(methyl)amino]methylidene}pentane-2,4-dione (3) Special details

**Table:**

Geometry. All esds (except the esd in the dihedral angle between two l.s. planes) are estimated using the full covariance matrix. The cell esds are taken into account individually in the estimation of esds in distances, angles and torsion angles; correlations between esds in cell parameters are only used when they are defined by crystal symmetry. An approximate (isotropic) treatment of cell esds is used for estimating esds involving l.s. planes.
Refinement. Refined as a 2-component twin.

### 3-{[(S)-Benzyl(methyl)amino]methylidene}pentane-2,4-dione (3) Fractional atomic coordinates and isotropic or equivalent isotropic displacement parameters (Å^2^)

**Table:**

	*x*	*y*	*z*	*U*_iso_*/*U*_eq_	
C1	1.1723 (4)	0.7494 (5)	0.1031 (3)	0.0494 (10)	
H1	1.2297	0.7243	0.1610	0.074*	
H2	1.1712	0.8689	0.0932	0.074*	
H3	1.2093	0.6937	0.0581	0.074*	
C2	1.0289 (3)	0.6899 (4)	0.0981 (2)	0.0307 (6)	
C3	0.9990 (3)	0.5183 (4)	0.1133 (2)	0.0300 (6)	
C4	1.1060 (4)	0.3913 (4)	0.1369 (2)	0.0364 (7)	
C5	1.0664 (4)	0.2163 (5)	0.1548 (3)	0.0455 (9)	
H4	1.1492	0.1519	0.1788	0.068*	
H5	1.0161	0.1655	0.1002	0.068*	
H6	1.0083	0.2183	0.1971	0.068*	
C6	0.8627 (3)	0.4704 (4)	0.1047 (2)	0.0309 (6)	
H7	0.8465	0.3569	0.1137	0.037*	
C7	0.6134 (3)	0.5100 (4)	0.0761 (2)	0.0315 (6)	
H9	0.6158	0.3879	0.0856	0.038*	
C8	0.5280 (3)	0.5433 (4)	−0.0157 (2)	0.0289 (6)	
C9	0.5840 (3)	0.6073 (4)	−0.0821 (2)	0.0335 (7)	
H10	0.6787	0.6336	−0.0698	0.040*	
C10	0.5044 (4)	0.6331 (5)	−0.1654 (2)	0.0373 (7)	
H11	0.5446	0.6774	−0.2096	0.045*	
C11	0.3657 (4)	0.5946 (5)	−0.1849 (2)	0.0379 (7)	
H12	0.3109	0.6116	−0.2421	0.045*	
C12	0.3084 (3)	0.5308 (4)	−0.1193 (2)	0.0349 (7)	
H13	0.2138	0.5040	−0.1319	0.042*	
C13	0.3882 (3)	0.5062 (4)	−0.0357 (2)	0.0310 (6)	
H14	0.3475	0.4635	0.0085	0.037*	
C14	0.5532 (4)	0.5888 (5)	0.1472 (2)	0.0408 (8)	
H15	0.4605	0.5463	0.1429	0.061*	
H16	0.5496	0.7089	0.1394	0.061*	
H17	0.6108	0.5619	0.2049	0.061*	
N1	0.7550 (3)	0.5668 (3)	0.08533 (19)	0.0329 (6)	
H8	0.7684	0.6724	0.0773	0.039*	
O1	0.9351 (3)	0.7936 (3)	0.07897 (18)	0.0389 (6)	
O2	1.2273 (3)	0.4204 (4)	0.1427 (2)	0.0560 (8)	
C15	0.5900 (4)	0.2575 (5)	0.4013 (3)	0.0407 (8)	
H18	0.5956	0.1369	0.4063	0.061*	
H19	0.6539	0.3075	0.4513	0.061*	
H20	0.6138	0.2921	0.3469	0.061*	
C16	0.4461 (3)	0.3127 (4)	0.4004 (2)	0.0326 (7)	
C17	0.4062 (3)	0.4854 (4)	0.3894 (2)	0.0297 (6)	
C18	0.4988 (3)	0.6153 (4)	0.3729 (2)	0.0327 (7)	
C19	0.4459 (4)	0.7895 (5)	0.3518 (3)	0.0454 (9)	
H21	0.4351	0.8439	0.4055	0.068*	
H22	0.3572	0.7854	0.3096	0.068*	
H23	0.5111	0.8518	0.3266	0.068*	
C20	0.2753 (3)	0.5311 (4)	0.3967 (2)	0.0302 (6)	
H24	0.2535	0.6452	0.3909	0.036*	
C21	0.0473 (3)	0.4873 (4)	0.4252 (2)	0.0316 (6)	
H26	0.0424	0.6097	0.4168	0.038*	
C22	0.0360 (3)	0.4507 (4)	0.5191 (2)	0.0280 (6)	
C23	0.1422 (3)	0.3812 (4)	0.5812 (2)	0.0323 (6)	
H27	0.2249	0.3525	0.5654	0.039*	
C24	0.1294 (4)	0.3531 (4)	0.6661 (2)	0.0367 (7)	
H28	0.2027	0.3040	0.7076	0.044*	
C25	0.0096 (4)	0.3967 (4)	0.6906 (2)	0.0364 (7)	
H29	0.0011	0.3790	0.7489	0.044*	
C26	−0.0973 (3)	0.4662 (4)	0.6291 (2)	0.0332 (7)	
H30	−0.1796	0.4955	0.6453	0.040*	
C27	−0.0847 (3)	0.4932 (4)	0.5438 (2)	0.0297 (6)	
H31	−0.1586	0.5408	0.5021	0.036*	
C28	−0.0694 (4)	0.4104 (5)	0.3571 (2)	0.0371 (7)	
H32	−0.1570	0.4518	0.3656	0.056*	
H33	−0.0663	0.2901	0.3635	0.056*	
H34	−0.0597	0.4400	0.2983	0.056*	
N2	0.1787 (3)	0.4311 (3)	0.4108 (2)	0.0341 (6)	
H25	0.1944	0.3242	0.4115	0.041*	
O3	0.3635 (3)	0.2058 (3)	0.4110 (2)	0.0472 (7)	
O4	0.6200 (2)	0.5899 (3)	0.37485 (18)	0.0418 (6)	

### 3-{[(S)-Benzyl(methyl)amino]methylidene}pentane-2,4-dione (3) Atomic displacement parameters (Å^2^)

**Table:**

	*U*^11^	*U*^22^	*U*^33^	*U*^12^	*U*^13^	*U*^23^
C1	0.032 (2)	0.0401 (19)	0.080 (3)	−0.0035 (15)	0.0196 (19)	−0.002 (2)
C2	0.0263 (15)	0.0337 (16)	0.0336 (15)	−0.0006 (12)	0.0100 (12)	−0.0033 (13)
C3	0.0267 (15)	0.0322 (15)	0.0324 (14)	0.0005 (12)	0.0096 (11)	−0.0028 (13)
C4	0.0285 (16)	0.0407 (18)	0.0424 (17)	0.0043 (14)	0.0132 (13)	0.0039 (15)
C5	0.039 (2)	0.0408 (19)	0.061 (2)	0.0109 (16)	0.0206 (18)	0.0113 (18)
C6	0.0273 (15)	0.0323 (15)	0.0336 (15)	0.0007 (12)	0.0077 (12)	0.0000 (13)
C7	0.0221 (14)	0.0322 (15)	0.0404 (16)	−0.0024 (12)	0.0074 (12)	0.0036 (14)
C8	0.0240 (14)	0.0258 (13)	0.0380 (15)	−0.0005 (11)	0.0093 (12)	−0.0014 (12)
C9	0.0254 (15)	0.0369 (16)	0.0394 (16)	−0.0027 (13)	0.0103 (12)	0.0002 (14)
C10	0.0348 (18)	0.0418 (18)	0.0366 (16)	−0.0046 (14)	0.0112 (14)	0.0006 (15)
C11	0.0349 (17)	0.0395 (17)	0.0367 (16)	−0.0039 (15)	0.0032 (13)	0.0003 (15)
C12	0.0267 (15)	0.0312 (15)	0.0459 (17)	−0.0056 (13)	0.0064 (13)	−0.0012 (14)
C13	0.0250 (14)	0.0281 (14)	0.0417 (16)	−0.0025 (12)	0.0113 (12)	−0.0009 (13)
C14	0.0330 (17)	0.053 (2)	0.0373 (16)	−0.0049 (16)	0.0109 (14)	−0.0009 (16)
N1	0.0231 (13)	0.0320 (13)	0.0425 (14)	−0.0017 (10)	0.0056 (11)	0.0029 (11)
O1	0.0307 (13)	0.0320 (12)	0.0555 (15)	0.0020 (10)	0.0129 (11)	0.0031 (11)
O2	0.0260 (13)	0.0546 (16)	0.089 (2)	0.0059 (12)	0.0179 (14)	0.0152 (16)
C15	0.0285 (17)	0.0375 (17)	0.059 (2)	0.0022 (14)	0.0153 (16)	−0.0033 (16)
C16	0.0283 (16)	0.0312 (15)	0.0410 (17)	−0.0016 (12)	0.0134 (13)	−0.0043 (14)
C17	0.0245 (14)	0.0317 (15)	0.0339 (14)	−0.0030 (12)	0.0091 (11)	−0.0019 (13)
C18	0.0267 (15)	0.0382 (16)	0.0345 (15)	−0.0058 (13)	0.0098 (12)	−0.0018 (14)
C19	0.0350 (19)	0.0384 (18)	0.067 (2)	−0.0026 (15)	0.0194 (17)	0.0120 (18)
C20	0.0262 (14)	0.0303 (14)	0.0357 (14)	−0.0023 (12)	0.0107 (12)	0.0010 (12)
C21	0.0227 (14)	0.0301 (15)	0.0442 (16)	0.0031 (12)	0.0123 (12)	0.0020 (14)
C22	0.0217 (13)	0.0239 (13)	0.0385 (15)	−0.0015 (10)	0.0075 (11)	−0.0019 (12)
C23	0.0242 (14)	0.0308 (15)	0.0409 (16)	0.0016 (12)	0.0055 (12)	−0.0029 (13)
C24	0.0342 (17)	0.0321 (15)	0.0389 (16)	0.0004 (13)	−0.0016 (14)	−0.0023 (14)
C25	0.0420 (19)	0.0315 (15)	0.0354 (15)	−0.0029 (14)	0.0085 (14)	−0.0047 (14)
C26	0.0314 (16)	0.0313 (15)	0.0400 (16)	−0.0008 (13)	0.0147 (13)	−0.0022 (14)
C27	0.0243 (14)	0.0277 (13)	0.0379 (15)	0.0013 (11)	0.0088 (11)	0.0005 (13)
C28	0.0285 (16)	0.0476 (19)	0.0355 (16)	0.0029 (14)	0.0079 (13)	−0.0009 (15)
N2	0.0254 (13)	0.0303 (13)	0.0510 (16)	0.0010 (10)	0.0175 (12)	0.0009 (12)
O3	0.0349 (14)	0.0309 (12)	0.082 (2)	−0.0018 (10)	0.0256 (14)	−0.0005 (13)
O4	0.0253 (11)	0.0447 (13)	0.0583 (15)	−0.0045 (10)	0.0152 (11)	0.0020 (13)

### 3-{[(S)-Benzyl(methyl)amino]methylidene}pentane-2,4-dione (3) Geometric parameters (Å, º)

**Table:**

C1—C2	1.504 (5)	C15—C16	1.510 (5)
C1—H1	0.9800	C15—H18	0.9800
C1—H2	0.9800	C15—H19	0.9800
C1—H3	0.9800	C15—H20	0.9800
C2—O1	1.246 (4)	C16—O3	1.236 (4)
C2—C3	1.453 (4)	C16—C17	1.455 (4)
C3—C6	1.399 (4)	C17—C20	1.396 (4)
C3—C4	1.472 (5)	C17—C18	1.468 (4)
C4—O2	1.224 (4)	C18—O4	1.228 (4)
C4—C5	1.517 (5)	C18—C19	1.518 (5)
C5—H4	0.9800	C19—H21	0.9800
C5—H5	0.9800	C19—H22	0.9800
C5—H6	0.9800	C19—H23	0.9800
C6—N1	1.311 (4)	C20—N2	1.321 (4)
C6—H7	0.9500	C20—H24	0.9500
C7—N1	1.470 (4)	C21—N2	1.463 (4)
C7—C8	1.522 (4)	C21—C28	1.524 (5)
C7—C14	1.527 (5)	C21—C22	1.534 (4)
C7—H9	1.0000	C21—H26	1.0000
C8—C9	1.394 (4)	C22—C23	1.387 (4)
C8—C13	1.399 (4)	C22—C27	1.400 (4)
C9—C10	1.383 (5)	C23—C24	1.388 (5)
C9—H10	0.9500	C23—H27	0.9500
C10—C11	1.392 (5)	C24—C25	1.392 (5)
C10—H11	0.9500	C24—H28	0.9500
C11—C12	1.390 (5)	C25—C26	1.387 (5)
C11—H12	0.9500	C25—H29	0.9500
C12—C13	1.385 (5)	C26—C27	1.392 (4)
C12—H13	0.9500	C26—H30	0.9500
C13—H14	0.9500	C27—H31	0.9500
C14—H15	0.9800	C28—H32	0.9800
C14—H16	0.9800	C28—H33	0.9800
C14—H17	0.9800	C28—H34	0.9800
N1—H8	0.8800	N2—H25	0.8800
			
C2—C1—H1	109.5	C16—C15—H18	109.5
C2—C1—H2	109.5	C16—C15—H19	109.5
H1—C1—H2	109.5	H18—C15—H19	109.5
C2—C1—H3	109.5	C16—C15—H20	109.5
H1—C1—H3	109.5	H18—C15—H20	109.5
H2—C1—H3	109.5	H19—C15—H20	109.5
O1—C2—C3	120.7 (3)	O3—C16—C17	121.0 (3)
O1—C2—C1	117.3 (3)	O3—C16—C15	117.5 (3)
C3—C2—C1	122.0 (3)	C17—C16—C15	121.5 (3)
C6—C3—C2	119.0 (3)	C20—C17—C16	119.0 (3)
C6—C3—C4	118.1 (3)	C20—C17—C18	118.2 (3)
C2—C3—C4	122.9 (3)	C16—C17—C18	122.8 (3)
O2—C4—C3	122.7 (3)	O4—C18—C17	122.8 (3)
O2—C4—C5	117.7 (3)	O4—C18—C19	117.2 (3)
C3—C4—C5	119.5 (3)	C17—C18—C19	120.1 (3)
C4—C5—H4	109.5	C18—C19—H21	109.5
C4—C5—H5	109.5	C18—C19—H22	109.5
H4—C5—H5	109.5	H21—C19—H22	109.5
C4—C5—H6	109.5	C18—C19—H23	109.5
H4—C5—H6	109.5	H21—C19—H23	109.5
H5—C5—H6	109.5	H22—C19—H23	109.5
N1—C6—C3	126.4 (3)	N2—C20—C17	126.4 (3)
N1—C6—H7	116.8	N2—C20—H24	116.8
C3—C6—H7	116.8	C17—C20—H24	116.8
N1—C7—C8	111.0 (3)	N2—C21—C28	109.8 (3)
N1—C7—C14	109.6 (3)	N2—C21—C22	111.0 (3)
C8—C7—C14	112.8 (3)	C28—C21—C22	112.3 (3)
N1—C7—H9	107.8	N2—C21—H26	107.8
C8—C7—H9	107.8	C28—C21—H26	107.8
C14—C7—H9	107.8	C22—C21—H26	107.8
C9—C8—C13	118.0 (3)	C23—C22—C27	118.6 (3)
C9—C8—C7	122.6 (3)	C23—C22—C21	122.6 (3)
C13—C8—C7	119.4 (3)	C27—C22—C21	118.7 (3)
C10—C9—C8	121.3 (3)	C22—C23—C24	121.0 (3)
C10—C9—H10	119.4	C22—C23—H27	119.5
C8—C9—H10	119.4	C24—C23—H27	119.5
C9—C10—C11	120.3 (3)	C23—C24—C25	120.1 (3)
C9—C10—H11	119.9	C23—C24—H28	119.9
C11—C10—H11	119.9	C25—C24—H28	119.9
C12—C11—C10	119.1 (3)	C26—C25—C24	119.4 (3)
C12—C11—H12	120.5	C26—C25—H29	120.3
C10—C11—H12	120.5	C24—C25—H29	120.3
C13—C12—C11	120.5 (3)	C25—C26—C27	120.4 (3)
C13—C12—H13	119.8	C25—C26—H30	119.8
C11—C12—H13	119.8	C27—C26—H30	119.8
C12—C13—C8	120.9 (3)	C26—C27—C22	120.4 (3)
C12—C13—H14	119.6	C26—C27—H31	119.8
C8—C13—H14	119.6	C22—C27—H31	119.8
C7—C14—H15	109.5	C21—C28—H32	109.5
C7—C14—H16	109.5	C21—C28—H33	109.5
H15—C14—H16	109.5	H32—C28—H33	109.5
C7—C14—H17	109.5	C21—C28—H34	109.5
H15—C14—H17	109.5	H32—C28—H34	109.5
H16—C14—H17	109.5	H33—C28—H34	109.5
C6—N1—C7	124.4 (3)	C20—N2—C21	124.0 (3)
C6—N1—H8	117.8	C20—N2—H25	118.0
C7—N1—H8	117.8	C21—N2—H25	118.0
			
O1—C2—C3—C6	1.2 (5)	O3—C16—C17—C20	4.2 (5)
C1—C2—C3—C6	−177.8 (3)	C15—C16—C17—C20	−174.5 (3)
O1—C2—C3—C4	−179.4 (3)	O3—C16—C17—C18	−177.2 (3)
C1—C2—C3—C4	1.6 (5)	C15—C16—C17—C18	4.1 (5)
C6—C3—C4—O2	177.2 (4)	C20—C17—C18—O4	172.0 (3)
C2—C3—C4—O2	−2.2 (5)	C16—C17—C18—O4	−6.7 (5)
C6—C3—C4—C5	−2.7 (5)	C20—C17—C18—C19	−8.3 (5)
C2—C3—C4—C5	177.8 (3)	C16—C17—C18—C19	173.1 (3)
C2—C3—C6—N1	−1.5 (5)	C16—C17—C20—N2	−1.8 (5)
C4—C3—C6—N1	179.0 (3)	C18—C17—C20—N2	179.5 (3)
N1—C7—C8—C9	4.7 (4)	N2—C21—C22—C23	3.2 (4)
C14—C7—C8—C9	128.1 (3)	C28—C21—C22—C23	126.6 (3)
N1—C7—C8—C13	−176.5 (3)	N2—C21—C22—C27	−178.7 (3)
C14—C7—C8—C13	−53.1 (4)	C28—C21—C22—C27	−55.3 (4)
C13—C8—C9—C10	−0.2 (5)	C27—C22—C23—C24	0.4 (5)
C7—C8—C9—C10	178.7 (3)	C21—C22—C23—C24	178.6 (3)
C8—C9—C10—C11	−0.3 (6)	C22—C23—C24—C25	−0.9 (5)
C9—C10—C11—C12	0.4 (6)	C23—C24—C25—C26	0.9 (5)
C10—C11—C12—C13	0.1 (5)	C24—C25—C26—C27	−0.4 (5)
C11—C12—C13—C8	−0.6 (5)	C25—C26—C27—C22	0.0 (5)
C9—C8—C13—C12	0.6 (5)	C23—C22—C27—C26	0.1 (5)
C7—C8—C13—C12	−178.3 (3)	C21—C22—C27—C26	−178.2 (3)
C3—C6—N1—C7	179.4 (3)	C17—C20—N2—C21	174.6 (3)
C8—C7—N1—C6	−119.2 (3)	C22—C21—N2—C20	−112.8 (3)
C14—C7—N1—C6	115.5 (4)	C28—C21—N2—C20	122.4 (3)

### 3-{[(S)-Benzyl(methyl)amino]methylidene}pentane-2,4-dione (3) Hydrogen-bond geometry (Å, º)

**Table:**

*D*—H···*A*	*D*—H	H···*A*	*D*···*A*	*D*—H···*A*
N1—H8···O1	0.88	1.94	2.597 (4)	131
N2—H25···O3	0.88	1.95	2.603 (4)	130
C14—H15···O2^i^	0.98	2.55	3.532 (5)	175
C14—H17···O4	0.98	2.66	3.482 (5)	142
C28—H32···O4^i^	0.98	2.54	3.512 (5)	173

Symmetry code: (i) *x*−1, *y*, *z*.
